# Transversus Abdominis Plane Block in Colorectal Surgery: A Meta-Analysis

**DOI:** 10.3389/fmed.2021.802039

**Published:** 2022-02-23

**Authors:** Dmitriy Viderman, Mina Aubakirova, Yerkin G. Abdildin

**Affiliations:** ^1^Department of Biomedical Sciences, Nazarbayev University School of Medicine (NUSOM), Nur-Sultan, Kazakhstan; ^2^Department of Mechanical and Aerospace Engineering, School of Engineering and Digital Sciences, Nazarbayev University, Nur-Sultan, Kazakhstan

**Keywords:** transversus abdominis plane (TAP) block, colorectal surgery, postoperative pain management, regional anesthesia, opioid consumption

## Abstract

Acute postoperative pain is one of the most common concerns during the early postoperative period in colorectal surgery. Opioids still represent the cornerstone of postoperative pain management, yet they often result in significant side effects such as nausea and/or vomiting, sedation, urinary retention, delayed recovery of colonic motility, respiratory depression, and postoperative ileus. Transversus abdominis plane (TAP) block has been widely used for postoperative analgesia in various abdominal surgeries. The primary aim of this meta-analysis was to compare the postoperative opioid requirements of patients in the TAP block group and the control group (placebo). The secondary aims included evaluation of the efficacy of TAP blocks in postoperative pain management, the measurement of time to first request for opioids, the measurement of length of hospital stay (LoS), and the documentation of postoperative nausea and/or vomiting. We searched for articles reporting the results of randomized controlled trials (RCTs) on the application of TAP block in colorectal surgery published before September 2021. Eight RCTs involving 615 patients were included in the meta-analysis. Seven articles reported the results of TAP blocks in laparoscopic surgery and eight in both laparoscopic and open surgery. The need for opioids and the intensity of pain at rest within 24 h after laparoscopic and combined (laparoscopic and open) surgeries were significantly lower in the TAP block group compared with the “no block” group. The intensity of pain during coughing within 24 hours after laparoscopic surgery was significantly lower in the TAP block groups compared to the groups without block. There were no statistically significant differences between the TAP block and “no block” groups in overall (over the entire hospital stay) postoperative opioid consumption and length of hospital stay after laparoscopic surgery, as well as in postoperative nausea and vomiting after laparoscopic and combined surgeries.

## Background

Colorectal surgery is one of the most frequently performed operations in abdominal surgery (1). Acute postoperative pain is one of the most common negative effects in the early postoperative period in colorectal surgery (2). Opioids still represent the cornerstone of postoperative pain management; however, they often result in significant side effects such as nausea and/or vomiting, sedation, urinary retention, delayed recovery of colonic motility, respiratory depression, and postoperative ileus (3, 4). Postoperative epidural analgesia had played an important role in postoperative pain management after colorectal surgery (5); however, it poses the risk of procedure-related complications (6). One of the options for the adequate management of postoperative pain in such surgeries is to use regional anesthetic techniques. The transversus abdominis plane (TAP) block has been widely used for postoperative analgesia in various abdominal surgeries. The TAP block is achieved through a direct blockade of the nerve afferent supplying the abdominal wall. TAP blocks target the ventral rami of intercostal nerves carrying pain fibers in the plane between the transversus abdominis and internal oblique muscles. Numerous studies have demonstrated that TAP blocks provide adequate analgesia and decrease postoperative opioid consumption after various operations including colorectal surgery (7), retropubic prostatectomy (8), cesarean delivery (9), abdominal hysterectomy (10), laparoscopic appendectomy, and incision hernia repair (11). The TAP block was introduced into clinical anesthesia to reduce postoperative pain and opioid consumption (12). However, meta-analyses of well-controlled studies focusing on TAP blocks in minimally invasive colorectal surgery are still lacking.

The main purpose of this meta-analysis was to compare the postoperative opioid requirements of patients with TAP blocks with patients within a control group (placebo). Secondary aims included the evaluation of the efficacy of TAP blocks in postoperative pain management, increase in time to first request for opioids, decrease in length of hospital stay (LoS), and decrease postoperative nausea and/or vomiting.

## Methods

### Protocol

We designed a protocol of the current systematic review (SR) with the inclusion and exclusion criteria for suitable articles. The SR protocol and methods of analysis were approved by all authors. We considered only randomized controlled trials (RCTs) that compared the analgesic effects of TAP block in colorectal surgery.

We followed the “Preferred Reporting Items for Systematic Reviews and Meta-Analyses (PRISMA)” (13) to prepare this systematic review.

We followed the PICO criteria:

Population: 18 years and older undergoing colorectal surgery (both open and laparoscopic).

Intervention: Transversus abdominis plane block.
Comparator: Placebo (sham).

Outcomes:

Primary – to assess opioid consumption within the first 24 h after surgery:
  a) In laparoscopic colorectal surgery only.
  b) In both laparoscopic and open.
Secondary – to assess the pain intensity scores following surgery; time to first request for rescue opioids; side effects of opioids (e.g., nausea and/or vomiting, pruritis respiratory depression); local anesthetic systemic toxicity (LAST), mechanical injury by the needle:
  a) In laparoscopic colorectal surgery only.
  b) In both laparoscopic and open.

### Inclusion Criteria

1) TAP block (both preoperative and postoperative) in acute pain management after colorectal surgery and standard non-interventional pain management methods assessed using the standard scales (VAS or NRS).
2) Randomized controlled trials (RCTs).
3) Age – 18 years old and older.
4) Colorectal surgery (both laparoscopic and open).

### Exclusion Criteria

We excluded studies that were not RCTs, which are case reports or series, editorials, cadaver studies, retrospective studies, technical reports.

### Search Methods

We performed a search for relevant articles in PubMed, Google Scholar, and the Cochrane Library published during the period from the inception of these databases to September 2021. The search included the following search terms or their combinations {[((“transversus abdominis plane block,”) “transversus abdominis plane”] “TAP block,”) “TAPB”} AND {[(“colorectal surgery”) “colon surgery”] OR “colon resection”}.

### Data Extraction and Statistical Methods

We calculated the sample mean and sample *SD* from data presented in 1st quartile, median, 3rd quartile, and sample size using the methods developed by Luo et al. (14) for the sample mean and by Wan et al. (15) for sample *SD*. We converted postoperative opioid doses into intravenous morphine equivalents (mg) to standardize outcome measures (16–18).

To convert fentanyl used in the reported studies to the equivalent morphine dose we used the following multiplier: 0.01. Doses reported in mcg were converted to mg dividing by 1,000. Data analysis was conducted using the Review Manager software (RevMan, version 5.4.1). Statistical heterogeneity was estimated by the I^2^ statistic.

### Assessment of Methodological Quality

The methodological quality of the RCTs was assessed independently by two reviewers using the Oxford quality scoring system [Jadad Scale (19)]. The quality of included studies was categorized within the range from 1 (min) to 5 (max) as low (<3), acceptable (3), good (4), and excellent (5).

## Results

**Figure 1:**
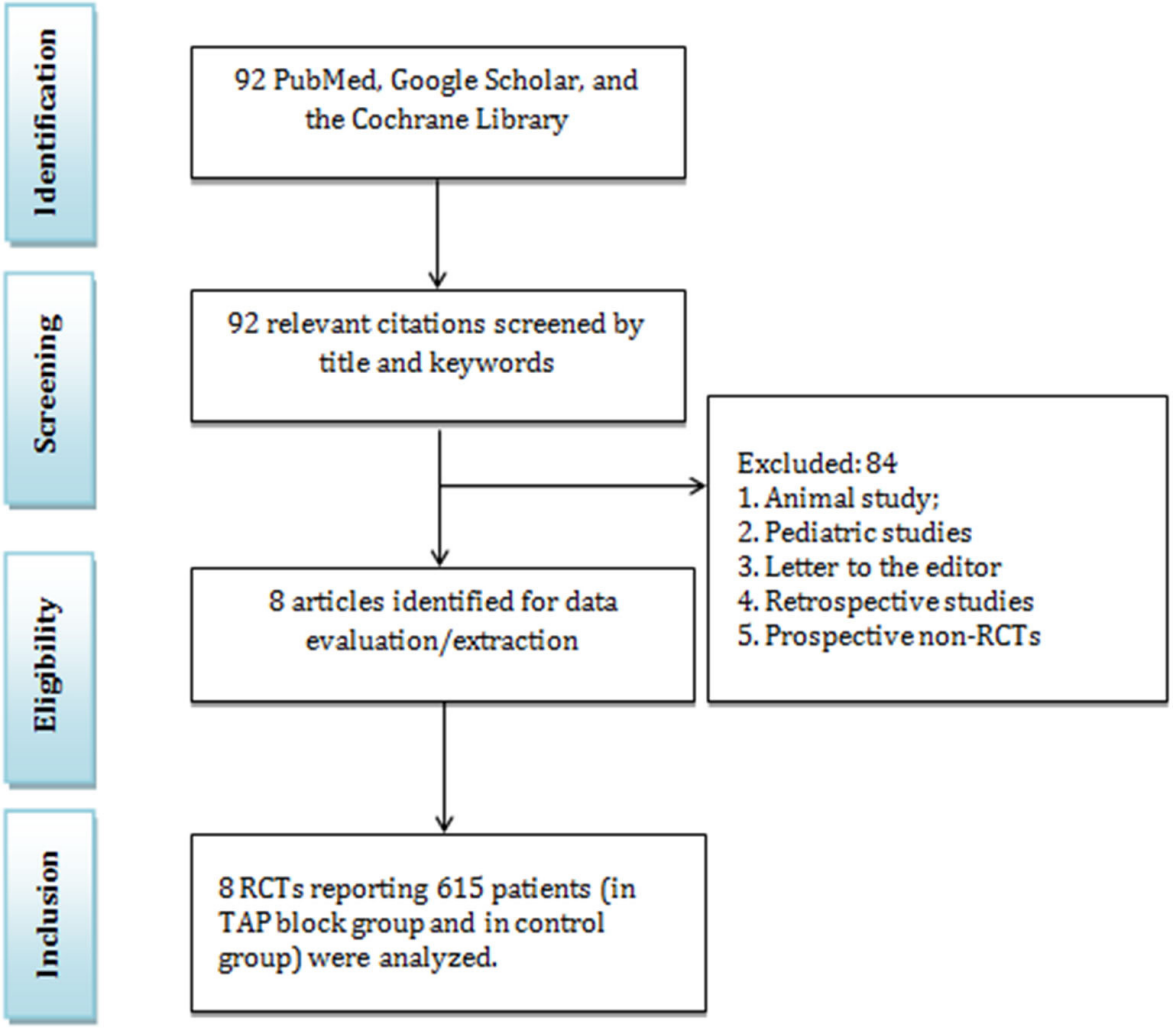
PRISMA diagram.

In this study, 92 articles were initially identified by the systematic search, in which 84 articles did not match the including criteria and were excluded (Figure 1). Among the remaining articles, 8 RCTs with 615 patients were included in the meta-analysis. Furthermore, 7 articles reported the results of TAP blocks in laparoscopic surgery and eight in both laparoscopic and open surgery. We analyzed the data related to postoperative opioid consumption, the efficacy of TAP blocks in the reduction of pain intensity, time to first need for opioids, the rate of postoperative side effects and complications in the TAP block group and “no block” group (Table 1). These studies were conducted in Thailand, the UK, the USA, South Korea, India, and Australia. In addition to the TAP block, the following analgesics were used: paracetamol, ketorolac, fentanyl (as PCA), flurbiprofenaxetil, sufentanil IV, morphine, hydromorphone, and oxycodone. The patient demographic data are presented in Table 1. There were no significant differences in age, ASA classification, and comorbidities. The most common types of colorectal surgeries included right hemicolectomy, left hemicolectomy, anterior resection, ileocolic and sigmoid resection (Table 2).

**Table 1:** Characteristics of studies included in the meta-analysis.

**1^**st**^ author, citation**	**Country**	**Study design**	**Study goals**	**Age (TAP/ intervention 2/Control, mean ± SD)**	**N of patients: total (TAP/intervention 2/Control)**	**Group**	**Surgery**	**General anesthesia**	**ASA status**	**Local anesthetics, volume and concentration, adjuvants**	**Postoperative analgesia**
Haruethaivijitchock et al. (20)	Thailand	RCT	Primary – fentanyl consumption; Secondary – pain scores, recovery outcome, and complications	65.44 ± 8.16 /64.19 ± 10.98	51 (25/26)	TAP: Modified continuous TAP C: Control	Laparoscopic colorectal surgery	Yes	I-III	TAP: 0.2% bupivacaine 5 ml bolus than 72 h infusion C: Normal saline	Oral paracetamol + IV-PCA: Fentanyl
Xu et al. (21)	China	RCT	Primary – hospital LOS Secondary – gastrointestinal motility, pain scores, plasma levels of cytokines	60.4 ± 9.3/ 61.4 ± 9.3/ 58.4 ± 10.4	165 (55/55/55) Patients in TEA group (*n =* 55) were not included in the meta-analysis	TAP: Single-shot bilateral subcostal and posterior TAP TEA (was not included in meta-analysis)	Laparoscopic colorectal cancer surgery	Yes	I-III	TAP: 2.5 mg/kg 0.375% ropivacaine	Flurbiprofen, for 48 h Rescue analgesia: Sufentanil; TAP: Infusion pump ropivacaine; GA: IV-PCA 1 μg/ml sufentanil, bolus 2 mL
Damadi et al. (22)	USA	RCT	Primary – total IV narcotic consumption Secondary – time to ambulation, time to bowel function, LOS	28.3/ 28.4/ 30.5	123 (41/51/31) Patients in ERP group (*n =* 51) were not included in the meta-analysis	TAP: TAP under laparoscopic visualization C: Control	Elective laparoscopic colorectal resection	NG	NG	TAP: 40 cc of 0.25% bupivacaine C: 40 cc of 0.9% normal saline	Fentanyl, morphine, or hydromorphone. Surgical wards: Tylenol PO e ibuprofen, + IV hydromorphone or
Oh et al. (23)	Korea	RCT	Primary – pain score on coughing on day 1 Secondary – pain at rest at all times and pain at coughing on days 2, 3	median: 66/65	55 (28/27)	TAP: US-guided TAP C: Control	Laparoscopic surgery for colorectal cancer	Yes	I-III	TAP: 0.5 mL/kg 0.25% bupivacaine C: 0.5 mL/kg normal saline	IV-PCA: Morphine 0.5 mg/mL and fentanyl 10 μg/mL
Smith et al. (24)	Australia	RCT	Primary – analgesic consumption Secondary – pain scores, respiratory function, PONV, hospital LOS, complications, patient satisfaction	64.82 ± 14.19/ 63.16 ± 14.49	142 (68/74)	TAP: US-guided bilateral TAP C: Standard care	Laparoscopic colorectal resectional surgery	Yes	I-IV	TAP: 3 mg/kg ropivacaine 40 mL (20 mL on each side) C: None	Paracetamol; PCA: Fentanyl 20 μg bolus,
Keller et al. (25)	USA	RCT	Pain scores, opioid use, PONV, short-term outcomes	67.34 ± 14.16/ 64.82 ± 13.11	77 (41/36)	TAP: TAP under laparoscopic visualization C: Control	Elective laparoscopic colorectal surgery	NG	I-IV	TAP: 0.5 ml/kg of 0.5% bupiva- caine, max 30 mL C: 0.5 ml/kg of 0.9% normal saline, max 30 mL	PCA morphine Nursing floor: Gabapentin + Toradol; Tylenol oral + oxycodone
Walter et al. (26)	UK	RCT	Primary – cumulative opioid use in 24 h Secondary – Opioid use at 2, 4, 6 h„ PONV, 30-day morbidity/mortality, LOS	64 ± 16.11/ 66 ± 12.24	68 (33/35)	TAP: Bilateral US-guided TAP C: Control	Elective laparoscopic colorectal resections	Yes	I-III	TAP: 40 mL 2 mg/kg levobupivacaine (150 mg max) C: No injection	IV paracetamol 1 g every 6 h PCA: Morphine 1 mg/mL bolus, lockout 5 min
Bharti et al. (27)	India	RCT	Pain scores, rescue analgesia consumption, time to first analgesia, cumulative morphine consumption, adverse effects	49.45 ± 13.29/ 42.20 ± 12.11	40 (20/20)	TAP: Bilateral TAP C: Control	Colorectal surgery via midline abdominal incision	Yes	I-III	TAP: 40 mL 0.25% bupivacaine C: 20 mL normal saline	IM diclofenac, Morphine

*ASA, American society of anesthesiologists; GA, general anesthesia; IV, intravenous; IM, intramuscular; LOS, length of stay; N, number; NG, not given; PACU, post-anesthesia care unit; PCA, patient-controlled analgesia; PO, postoperative; PONV, postoperative nausea and vomiting; RCT, randomized controlled trial; SD, standard deviation; TAP, transversus abdominis plane; TEA, Thoracic epidural anesthesia; US-guided, ultrasound-guided; VAS, visual analog scale*.

**Table 2:** Distribution of the types of colorectal surgeries.

**First author, year**	**Type of surgery**	**TAP block, *n***	**TAP block, % of 317**	**Control, *n***	**Control, % of 322**
Haruethaivijitchock et al. (20)	Right hemicolectomy	10	3.2%	11	3.4%
	Left hemicolectomy	7	2.2%	4	1.2%
	Anterior resection	5	1.6%	7	2.2%
	Sigmoid resection	0	0.0%	2	0.6%
	Subtotal colectomy	3	0.9%	12	3.7%
Xu et al. (21)	Right hemicolectomy	23	7.3%	21	6.5%
	Left hemicolectomy	10	3.2%	8	2.5%
	Anterior resection	16	5.0%	20	6.2%
	Sigmoid resection	11	3.5%	11	3.4%
Damadi et al. (22)	Anterior resection	4	1.3%	6	1.9%
	Ileocolic/sigmoid resection (non-specified)	30	9.5%	25	7.8%
	Abdominal perineal resection	3	0.9%	0	0.0%
	Total abdominal colectomy	2	0.6%	0	0.0%
	Total proctocolectomy	0	0.0%	0	0.0%
	Colostomy reversal	2	0.6%	0	0.0%
Oh et al. (23)	Colon (non-specified)	11	3.5%	18	5.6%
	Rectum (non-specified)	17	5.4%	9	2.8%
Smith et al. (24)	Right hemicolectomy	29	9.1%	34	10.6%
	Left hemicolectomy	3	0.9%	5	1.6%
	Anterior resection	33	10.4%	35	10.9%
	Subtotal colectomy	4	1.3%	0	0.0%
Keller et al. (25)	Resection rectopexy	1	0.3%	1	0.3%
	Anterior resection	3	0.9%	8	2.5%
	Ileocolic/sigmoid resection (non-specified)	32	10.1%	27	8.4%
	Abdominal perineal resection	1	0.3%	1	0.3%
	Total abdominal colectomy	3	0.9%	1	0.3%
	Total proctocolectomy	1	0.3%	1	0.3%
Walter et al. (26)	Right hemicolectomy	14	4.4%	14	4.3%
	Left and rectal resection (non-specified)	19	6.0%	21	6.5%
Bharti et al. (27)	Not mentioned	20	6.3%	20	6.2%
	**Total**	**307**	**100%**	**322**	**100%**
**Types of operations sorted by the number of patients in descending order**	Right hemicolectomy	76	24.0%	80	24.9%
	Ileocolic/sigmoid resection (non-specified)	62	19.6%	52	16.2%
	Anterior resection	61	19.2%	76	23.7%
	Not mentioned	20	6.3%	20	6.2%
	Left hemicolectomy	20	6.3%	17	5.3%
	Left and rectal resection (non-specified)	19	6.0%	21	6.5%
	Rectum (non-specified)	17	5.4%	9	2.8%
	Colon (non-specified)	11	3.5%	18	5.6%
	Sigmoid resection	11	3.5%	13	4.0%
	Subtotal colectomy	7	2.2%	12	3.7%
	Total abdominal colectomy	5	1.6%	1	0.3%
	Abdominal perineal resection	4	1.3%	1	0.3%
	Colostomy reversal	2	0.6%	0	0.0%
	Total proctocolectomy	1	0.3%	1	0.3%
	Resection rectopexy	1	0.3%	1	0.3%

*Table 2 shows the distribution of types of colorectal surgery in RCTs in our meta-analysis. Right hemicolectomy, ileocolic/sigmoid resection (non-specified), and anterior resection are the three main types of surgery that were used together in 62.8 and 64.8% of patients in the TAP block and control groups, respectively*.

### Volume, Dose, and Concentration of Local Anesthetics

Three local anesthetics were used for TAP block (Table 1): ropivacaine (in 3 studies), bupivacaine (in 5 studies), and levobupivacaine (in 1 study). The authors reported using a concentration and dose of ropivacaine from 0.375%−2.5 mg/kg (in 2 studies) to 0.375%−3 mg/kg−20 ml on each side (in 1 study). The concentration and dose of bupivacaine varied from 0.2% (given as a 5 ml bolus and continued as an infusion for 72 h) to 0.5 mL/kg of.5% bupivacaine, maximum volume - 30 ml. Levobupivacaine was given as 40 mL 2 mg/ levobupivacaine (150 mg max).

### Opioid Consumption Within 24 h After Surgery (in mg of Morphine)

Three studies in our meta-analysis (22, 26, 27) reported the total morphine consumption in mg, and one (20) in fentanyl in mcg delivered via PCA which we converted into morphine consumption in mg. Walter et al. (26) reported data for open surgery and laparoscopic colorectal surgery. They also reported the postoperative opioid consumption separately for laparoscopic colorectal surgery (LCS), so we used that data to keep consistency with other studies. Bharti et al. (27) conducted colorectal surgery via midline abdominal incision.

One study (25) reported the opioid consumption (morphine equivalent) in the postanesthesia care unit (PACU) and using the Defined Daily Dose (DDD), but since we consider values at 24 h after surgery, we could not include this study in our meta-analysis. Another study (23) reported the postoperative analgesic consumption in mL and it was not clear which opioid was used, so we were unable to include that study results in our analysis.

**Figure 2:**
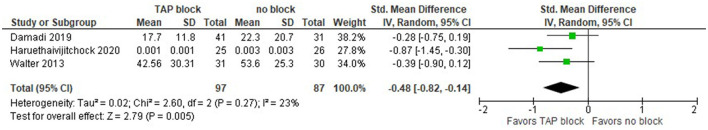
Forest plot for total opioid consumption within 24 h after surgery in mg of morphine (only laparoscopic surgeries).

Therefore, only three studies provided data on postoperative opioid consumption for laparoscopic surgery. The opioid consumption within 24 h after surgery is presented in the forest plot (Figure 2). Although the overall effect favors TAP block over no block (control group), confidence intervals cross zero in two studies. Due to different types of opioids used in the studies, we constructed the model with the random effects analysis and the standardized mean difference for the effect measure; the standardized mean difference with 95% CI is as follows: −0.48 [−0.82, −0.14]. Since the studies have similar sample sizes, the weights of the studies are approximately equal (~28–38%). The total number of patients in the TAP block groups is 97, while in the control groups there are 87 patients. The value of I^2^ is equal to 23%, the model shows heterogeneity, so we performed the sensitivity analysis by excluding one study at a time. The model is sensitive to the results of two studies (20, 26), in which case the model is indifferent between TAP block and no block.

**Figure 3:**
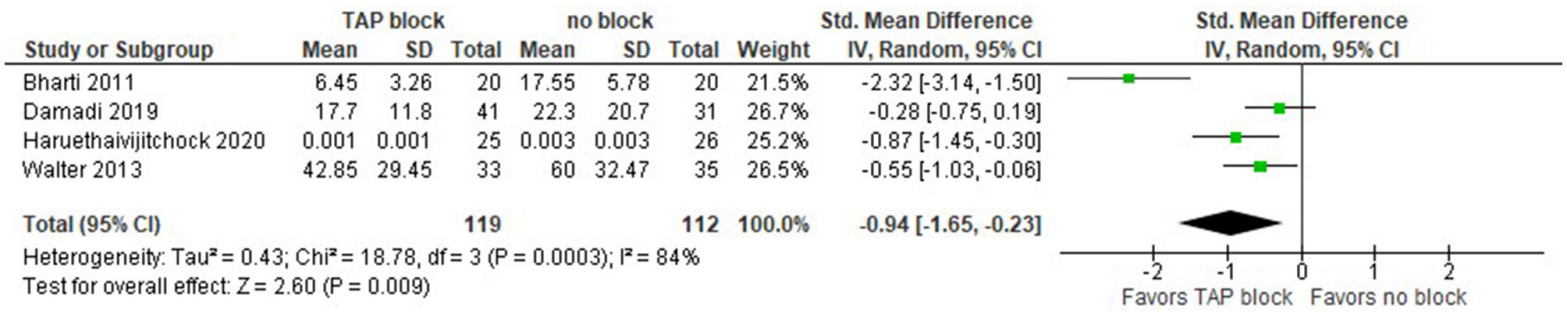
Forest plot for total opioid consumption within 24 h after surgery in mg of morphine (laparoscopic and open surgeries).

Afterward, we included both laparoscopic and open surgeries, namely, colorectal surgery *via* midline abdominal incision like in Bharti et al. Walter et al. (26) presented data for open and laparoscopic surgeries, as well as laparoscopic only. The results slightly changed. The opioid consumption within 24 h after surgery is presented in the forest plot in Figure 3. The model still favors TAP block over no block (control group), the standardized mean difference with 95% CI is now: −0.94 [−1.65, −0.23]. Since the studies have similar sample sizes, the weights of the studies are approximately equal (~22–27%). The total number of patients in the TAP block groups is now 119, while in the control groups there are 112 patients. The value of I^2^ is equal to 84%, so the model shows high heterogeneity and this is significant since the *P*-value is equal to 0.0003. Due to the high heterogeneity of the studies, we performed the sensitivity analysis by excluding one study at a time. The model is sensitive to the results of a study by Haruethaivijitchock et al. (20).

### Overall Postoperative Opioid Consumption (in mg of Morphine)

**Figure 4:**
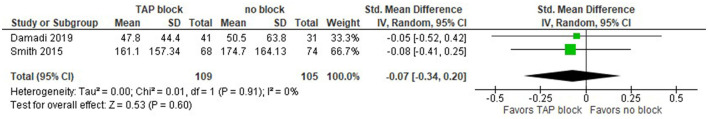
Forest plot for overall postoperative opioid consumption in mg of morphine (laparoscopic surgeries).

Only three studies reported the overall postoperative opioid consumption, but one of them (20) did not provide complete data values, so we were unable to include them in this analysis. Smith et al. (24) reported data in terms of the sample mean and SEM, so we estimated the sample *SD* as SEM^*^SQRT(*n*), where *n* is the sample size. This, however, makes the *SD* considerably larger than in other studies, e.g., in Damadi et al. (22). As we can see, the overall effect does not favor TAP block over the no block alternative, but with a larger number of studies, we could have received clearer results (Figure 4).

### Pain Intensity in NRS/VAS Scores at Rest Recorded 24 h After Surgery

**Figure 5:**
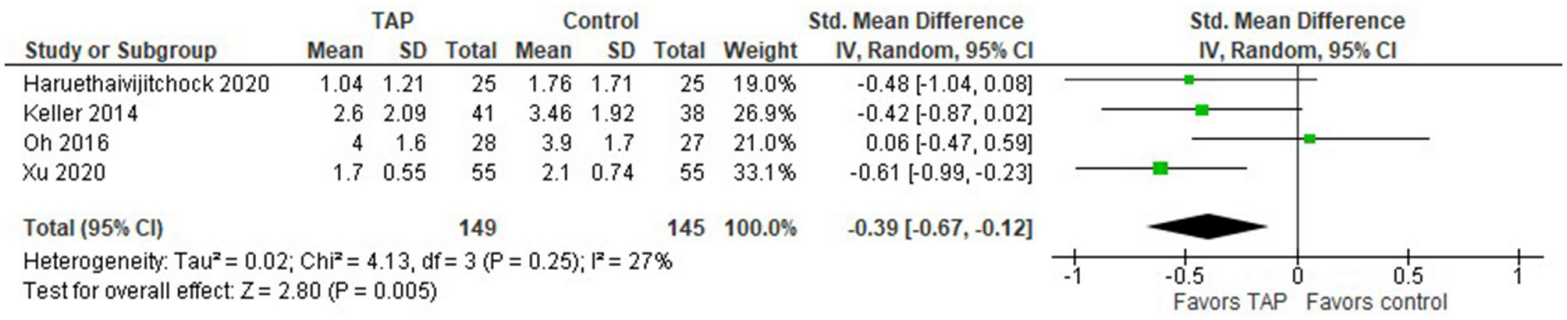
Forest plot for the pain intensity score in NRS/VAS at rest recorded 24 h after laparoscopic surgery.

The pain intensity measured in NRS/VAS scores at rest recorded 24 h after laparoscopic surgery is presented in the forest plot (Figure 5). The model includes four studies (20, 21, 23, 25). In this model, we used the standardized mean difference as a summary statistic because all studies measure the same outcome (pain intensity score), but measure it using different scales (NRS/VAS). In this analysis, there are 149 patients in the TAP groups and 145 patients in the control groups. Since the number of patients in each study is of the same order, the model assigns comparable weights for each study (from 19 to 33.1%).

This model shows that the patients tend to have less pain intensity after laparoscopic surgery when TAP block was applied compared to the patients in the control group, the standardized mean difference with 95% CI is as follows: −0.39 [−0.67, −0.12]. This result is not strong though, because the confidence intervals cross zero in three studies. The sensitivity analysis showed that the result is sensitive to the exclusion of one study at a time. In addition, Bharti et al. (27) found that “TAP group patients had significantly lower pain scores at rest,” but they provided only a graphical representation of their result and we were unable to include their result in our meta-analysis. Considering 20 patients in both TAP and control groups in their study, their result would add additional points to the TAP block.

**Figure 6:**
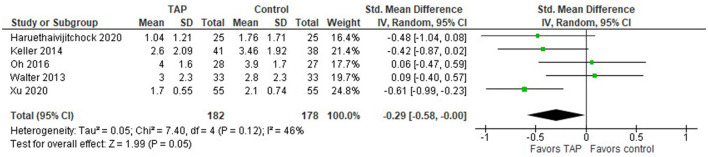
Forest plot for the pain intensity score in NRS/VAS at rest recorded 24 h after laparoscopic and open surgeries.

When both laparoscopic and open surgeries are considered, the model (Figure 6) still favors TAP block over the no block option, but the overall effect becomes closer to the no-difference point, the standardized mean difference with 95% CI is as follows: −0.29 [−0.58, −0.00].

### Pain Intensity Score in NRS/VAS When Coughing at 24 h After Surgery

**Figure 7:**
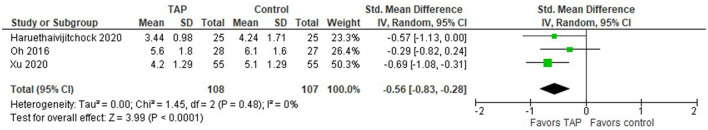
Forest plot for the pain intensity score in NRS/VAS when coughing recorded 24 h after open and laparoscopic surgeries.

Three studies in our analysis compared TAP block with no block management in terms of the pain intensity scores when patients are coughing. The forest plot in Figure 7 favors the TAP block when coughing at the time 24 h after the surgery. The model is not sensitive to the exclusion of any study, the summary effect does not change significantly. No heterogeneity was observed across studies.

### Length of Hospital Stay (Days)

**Figure 8:**
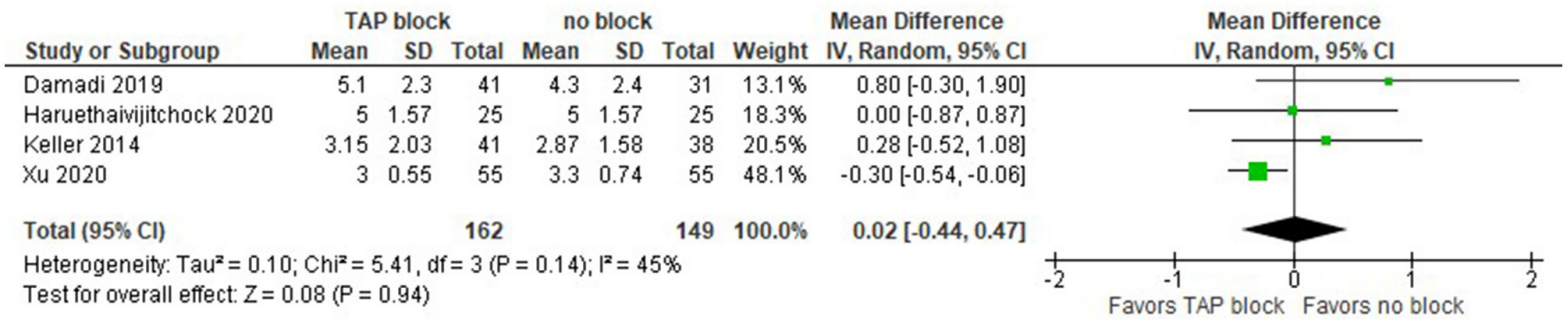
Forest plot on length of hospital stay after laparoscopic surgery.

An interesting result is observed in terms of the length of hospital stay (LOS) after laparoscopic surgery. The meta-analysis shows no difference between TAP block and non-block options (Figure 8). In studies by Damadi et al. (22) and Keller et al. (25), the LOS was shorter in the control group than in the TAP block group, while in a study by Haruethaivijitchock et al. (20) there was no difference.

We should mention here that in the latter study, the data were presented as mean (5) and IQR (4.6), so we assumed that the authors meant that (4.6) are the 1^st^ and 3^rd^ quartiles. One study did not report the sample *SD*; we were unable to incorporate its result into the forest plot. In particular, Smith et al. (24) reported the sample mean (7.5 vs 6.3) and median (4 for both) of LOS for TAP block and control groups.

### Postoperative Side Effects (Nausea and Vomiting)

**Figure 9:**
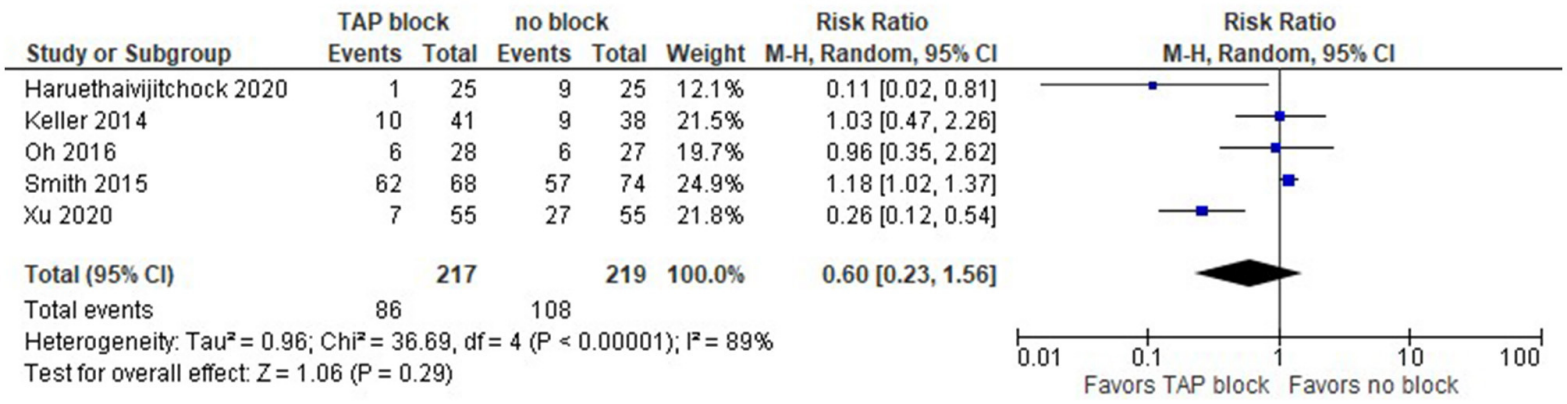
Forest plot on postoperative nausea and vomiting (laparoscopic surgery).

Postoperative side effects (nausea and vomiting) from opioids in the TAP and control groups are depicted in the forest plot below for laparoscopic surgery (Figure 9). The summary effect of the model shows no difference between TAP block and non-block options (risk ratio with 95% CI: 0.60 [0.23,1.56]), and the result is insensitive to the exclusion of any study.

We should mention that since Smith et al. (24) did not provide the overall number of patients in PONV, we used the values for PONV 24 h after surgery, which were the largest values in their reports among PONV values at 24, 48, and 72 h. Xu et al. (21) reported the mean values of the PONV case, not the actual number of patients, but we assume that the sample mean could be a reasonable estimate for this analysis.

**Figure 10:**
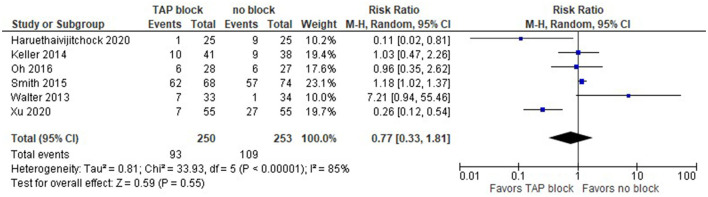
Forest plot on postoperative nausea and vomiting (laparoscopic and open surgeries).

The model (Figure 10) shows no difference between TAP block and no block when we consider both laparoscopic and open surgeries (risk ratio with 95% CI: 0.77 [0.33, 1.81]). This result is not sensitive to the exclusion of any study.

### Assessment of Methodological Quality (Jadad/Oxford Quality Scoring System)

Further results showed that six out of eight studies were graded as excellent (5/5 points), one – as good (4/5 points), and one as acceptable (3/5). The grading of the included studies is presented in Table 3.

**Table 3:** Oxford quality scoring system (Jadad Scale).

**First author, year**	**Was the study described as randomized?**	**Was the method used to generate the sequence of randomization described and appropriate?**	**Was the study described as double blind?**	**Was the method of double blind described and appropriate?**	**Was there a description of withdraw and dropouts?**	**Total score**
Haruethaivijitchock et al. (20)	1	1	1	1	1	5
Xu et al. (21)	1	1	0	0	1	3
Damadi et al. (22)	1	1	1	1	1	5
Oh et al. (23)	1	1	1	1	1	5
Smith et al. (24)	1	1	1	1	1	5
Keller et al. (25)	1	1	1	1	1	5
Walter et al. (26)	1	1	1	1	1	5
Bharti et al. (27)	1	1	1	1	0	4

*The table represents an independent assessment of methodological quality of all studies included in the meta-analysis*.

## Discussion

This meta-analysis reports the evidence on the clinical position of the TAP block in pain management after laparoscopic colorectal surgery. TAP block was found to decrease the cumulative opioid requirement within 24 h after surgery in both groups: laparoscopic alone and combined (laparoscopic and open). However, the effect of TAP block in reducing opioid requirements was more pronounced in the combined group. It can be explained by a more extensive surgical incision in open colorectal surgery. Therefore, TAP block was more clinically useful in patients after open surgery, and compared to “no block”, patients who received TAP block required much fewer opioids. Nevertheless, there was no statistically significant difference in overall opioid requirement after laparoscopic surgery. TAP block was superior to “no block” in reducing the pain intensity at rest and on coughing within 24 h after surgery in both groups: laparoscopic alone and combined (laparoscopic and open). There were no statically significant differences in length of hospital stay. There were no statistically significant differences between the TAB block and no block in the incidence of postoperative nausea and vomiting in both groups: laparoscopic alone and combined (laparoscopic and open).

Colorectal surgery is among the most frequently performed types of abdominal surgeries. Nowadays, there is a tendency toward shifting from open to laparoscopic surgeries. Minimally invasive colorectal surgery is the current standard of surgical care. The laparoscopic technique improves patient outcomes and reduces cost-effectiveness (28, 29). Laparoscopic colorectal procedures have been shown to enhance the return of bowel function (30).

An ultrasound-guided TAP block functions by blocking the intercostal nerve running from the spinal nerve root to result in analgesia (7). TAP block has been consistently reported to result in opioid requirements. Thus, previous studies reported up to a 70% reduction in morphine consumption after colorectal surgery in patients with TAP block using 20 ml of 0.375% levobupivacaine (7). In another study, TAP block using 0.75% ropivacaine significantly decreased postoperative morphine consumption during 48 h following surgery and prolonged the time to the first analgesic request after abdominal hysterectomy (27). The early postoperative period in major surgeries is a critically important period that can be associated with complications such as respiratory depression, myocardial infarction, and hypotension. Adequate postoperative pain management and reduction in opioid consumption can reduce these complications, especially in elderly morbid patients.

Well-controlled postoperative pain has been associated with improvement in early mobilization, patient satisfaction, shortened hospital stay, reduced hospital costs, and overall improved outcomes, TAP block compared to patient-controlled analgesia found a significant decrease in intravenous opioid use a trend toward a shorter length of hospital stay (31). In some cases, TAP block fails to produce adequate analgesia. One explanation for that is not adequate coverage to achieve blockage of sensory dermatomes of the entire region in colorectal surgery. It has been reported in most of the cadaveric studies that ultrasound-guided TAP block covers T10–L1, the region localized below the umbilicus (11). Therefore, TAP can be effectively used in retropubic radical prostatectomy, hernia repair, total abdominal hysterectomy, exploratory laparotomy, and cesarean delivery (12–14). Previous studies showed that the trocar insertion sites localized above the umbilicus in the upper quadrants (T8–9 dermatome) are not covered by the TAP block (32, 33). Therefore, localization of trocar insertion sites in an area that is partially covered or not covered by the TAP block could result in negative results. Since there are no major nerve bundles and the innervation is indistinct in the block region, the successful implementation of TAP block depends on the volume and spread of the local anesthetic solution within the anatomical plane. Ultrasound-guided TAP block is a relatively safe procedure, and previous studies have not found serious complications associated with this procedure (8, 12, 16, 34, 35).

The ultrasound guidance improves visualization of the layers of the abdominal wall and allows safe needle placement and anesthetic injection in the correct plane.

## Limitations

The main limitations of this meta-analysis are the inclusion of single centered studies and relatively small sample sizes. Since the studies had strict inclusion and exclusion criteria, they might not be representative of real-world evidence as well as patients in other countries or even other medical centers. Another possible limitation is that medical and research staff (anesthesiologists, surgeons, investigators) and patients were not blinded, therefore, these factors could also add a bias. There was also the heterogeneity among these studies in terms of timing of TAP block (preoperative vs. postoperative), reporting format of the variables, the timing of the assessment of pain severity, local anesthetics, and their volumes and concentrations, single-shot vs. continuous administration of local anesthetics, different technical modifications of TAP block.

## Conclusions

This meta-analysis showed that opioid requirement within 24 h after surgery as well as pain intensity at rest within 24 h after laparoscopic and combined (laparoscopic and open) types of surgeries were significantly lower in the TAP block groups compared to “no block” groups. The intensity of pain during coughing within 24 hours after laparoscopic surgery was significantly lower in the TAP block groups compared to the groups without block. However, there were no statistically significant differences between the TAP block and “no block” groups in overall (i.e., within and after 24 h) postoperative opioid consumption and length of hospital stay after laparoscopic surgery, as well as in postoperative nausea and vomiting after laparoscopic and combined surgeries. Since a limited number of publications and limited quality of evidence is currently available, more high-quality randomized controlled trials are required.

## Data Availability Statement

The original contributions presented in the study are included in the article/supplementary material, further inquiries can be directed to the corresponding author/s.

## Author Contributions

DV conceived the idea for this paper, wrote the protocol, conducted the literature review, and contributed to writing. MA extracted the data and edited the manuscript. YA conducted a statistical analysis and contributed to writing and editing. All authors contributed to the article and approved the submitted version.

## Funding

This meta-analysis was supported by the Nazarbayev University Faculty Development Competitive Research Grant 2021–2023. Funder project reference: 021220FD2851.

## Conflict of Interest

The authors declare that the research was conducted in the absence of any commercial or financial relationships that could be construed as a potential conflict of interest.

## Publisher's Note

All claims expressed in this article are solely those of the authors and do not necessarily represent those of their affiliated organizations, or those of the publisher, the editors and the reviewers. Any product that may be evaluated in this article, or claim that may be made by its manufacturer, is not guaranteed or endorsed by the publisher.
